# Medial patellofemoral ligament reconstruction combined with biplanar supracondylar femoral derotation osteotomy in recurrent patellar dislocation with increased femoral internal torsion and genu valgum: a retrospective pilot study

**DOI:** 10.1186/s12891-021-04816-2

**Published:** 2021-11-26

**Authors:** Xiangtian Deng, Lingzhi Li, Peng Zhou, Fuyuan Deng, Yuan Li, Yanwei He, Ge Chen, Zhong Li, Juncai Liu

**Affiliations:** 1 School of Medicine, Nankai University, Tianjin, 300071 People’s Republic of China; 2 Department of Orthopaedic Surgery, The Affiliated Hospital of Southwest Medical University, Lu Zhou, 646000 Sichuan Province People’s Republic of China; 3 Sichuan Provincial Laboratory of Orthopaedic Engineering, Department of Bone and Joint Surgery, The Affiliated Hospital of Southwest Medical University, Lu Zhou, 646000 Sichuan Province People’s Republic of China

**Keywords:** Recurrent patellar dislocation, Genu valgum, Femoral anteversion angle, Femoral derotation osteotomy, Biplanar, Medial patellofemoral ligament reconstruction, Alignment correction

## Abstract

**Background:**

The purpose of this study was to evaluate the clinical and radiographic outcomes after medial patellofemoral ligament (MPFL) reconstruction combined with supracondylar biplanar femoral derotation osteotomy (FDO) in recurrent patellar dislocation (RPD) with increased femoral anteversion angle (FAA) and genu valgum.

**Methods:**

Between January 2017 to December 2020, a total of 13 consecutive patients (13 knees, 4 males and 9 females, mean age 18.7 (range, 15–29 years) with RPD with increased FAA (FAA > 25°) and genu valgum (mechanical axis deformity of ≥5°) who underwent supracondylar biplanar FDO using a Tomofix-locking plate combined with MPFL reconstruction in our institution were included. Preoperative full-leg standing radiographs, lateral views, and hip-knee-ankle computed tomography (CT) scans were used to evaluate the mechanical lateral distal femoral angle (mLDFA), anatomical femorotibial angle (aFTA), mechanical axis, patellar height, tibial tubercle-trochlear groove (TT-TG) distance, and torsional angle of the tibial and femoral in the axial plane. Patient reported outcomes were evaluated using the International Knee Documentation Committee (IKDC) score, Kujala score, Lysholm score, visual analog scale (VAS), and Tegner score preoperatively and postoperatively. Postoperative CT scans were used to evaluate the changes of FAA and TT-TG, and full-leg standing radiographs was used to evaluate the changes of mLDFA, aFTA, and mechanical axis.

**Results:**

A total of 13 patients (13 knees) were included with an average follow-up period of 26.7 months (range 24–33). No cases developed wound infection, soft tissue irritation, and recurrent patellar dislocation during the follow-up period after surgery. Bone healing at the osteotomy site was achieved in all cases, and all patients regained full extension and flexion. Clinical outcomes (VAS, Kujala, IKDC, Lysholom, and Tegner scores) improved significantly at the final follow-up after surgery (*p* < 0.05). The mean mLDFA, aFTA, mechanical axis, and TT-TG distance showed statistically significant improvement following the combined surgery (*p* < 0.05), while the CDI did not change significantly after surgery (*p*>0.05).

**Conclusion:**

MPFL reconstruction combined with supracondylar biplanar FDO showed satisfactory clinical outcomes and radiographic results in the short-term follow-up period.

## Background

Recurrent patellar dislocation (RPD) is a complex condition, and multiple contributing factors for patellar instability have been identified, including patella alta, genu valgum, disrupted or weaken medial soft tissue, trochlear dysplasia, increased tibial tuberosity and the trochlear groove (TT-TG) distance, and rotational malalignment of the femur or tibia [1–5]. Specially, osseous deformities in the coronal and axial plane, such as genu valgum and torsional deformities of the lower extremity, are now considered to be associated with adverse effects on patellofemoral instability [6, 7]. Herein, the increased femoral anteversion angle (FAA) and genu valgum are thought to create a sustaining lateralizing force vector applied on the patella, which might increase excessive loading forces on the reconstructed graft and even lead to patellar redislocation [8, 9].

At present, there is still much controversy in the surgical techniques of RPD combined with knee valgus deformity. In recent years, various surgical techniques for addressing RPD with genu valgum have been described [10–12]. Generally, the MPFL is the major stabilizer which restricts lateral patellar displacement from zero to thirty of knee flexion, and MPFL reconstruction has been validated as a reliable surgical procedure for treating recurrent patellar instability. Nevertheless, isolated MPFL reconstruction might not be sufficient in patients with increased femoral anteversion and genu valgum, as it does not address the underlying lateralizing force vector acting on the patella [13].

Although it has been shown that genu valgum combined with excessive femoral internal torsion are primary risk factors for RPD, it is rarely corrected by surgery simultaneously. Despite studies that confirm the association between mechanical malalignment of multi-plane and patellar instability, there is a paucity of studies published regarding clinical and radiographic results after MPFL reconstruction combined with supracondylar biplanar FDO procedure in this population.

The purpose of this study was to a) analyze the clinical and radiographic results of the MPFL reconstruction combined with biplanar supracondylar FDO procedure, b) to evaluate the differences between pre- and post-operative knee function and radiographic results including effects on patellar parameters and alignment correction, c) to assess complications associated with bone healing, soft tissue irritation, wound infection, and recurrence of dislocation. It was our hypothesis that patients with RPD associated with increased FAA and genu valgum treated with biplanar supracondylar FDO and MPFL construction can prevent patellar re-dislocation, achieve satisfactory clinical and radiographic results in the short-term follow-up period.

## Methods and methods

### Patients

This study was approved by the Ethics Committee of the Affiliated Hospital of Southwest University (ID: 2016–108) and was performed in accordance with the Declaration of Helsinki. Between January 2017 and December 2020, a retrospective pilot study was conducted to evaluate the clinical and radiographic outcomes of patients who experienced recurrent patellar instability due to genu valgum in the coronal plane and increased FAA in the axial plane. All included patients were treated by MPFL reconstruction combined with supracondylar biplanar FDO procedure and all surgeries were performed by the same senior orthopaedic surgeon (Z.L).

Inclusion criteria were as follows: a) recurrent patellar dislocations (≥2 times); b) increased FAA ≥25°; c) genu valgum (mechanical axis deformity of ≥5°); d) physiologic trochlea and/or Dejour type A dysplasia [14]; e) patients with a minimum postoperative follow-up of 2-years.

Exclusion criteria were as follows: a) tibial rotational deformity (> 30°); b) patients with open growth plates; c) patients with lateral compartment knee osteoarthritis; d) patients with previous knee surgery; e) posttraumatic deformities; f) patients who lost to follow up; g) incomplete clinical data.

### Surgical technique

#### Supracondylar biplanar femoral derotation osteotomy

Supracondylar biplanar FDO was performed according to a method described previously by Hinterwimmer et al. [15], in which they developed an anterior closed-wedge technique of biplanar supracondylar DFO for patellofemoral malalignment. Patients received general anesthesia and were placed on a radiolucent operating table in a supine position, and a tourniquet was used to the proximal thigh. First, an arthroscopic examination was performed at the beginning of surgery to evaluate the patellar tracking under the direct visualization.

Second, all cases were performed by the standard medial subvastus approach through a longitudinal skin incision of approximately 10–12 cm in length to access the medial metaphysis of the distal femur. The subcutaneous tissue and fascia were separated and the vastus medialis was stripped with a blunt Hohmann retractor to protect the neurovascular behind the femoral shaft. The axial osteotomy was performed perpendicular to the femoral shaft axis and involved the posterior two-thirds of the femur. The frontal plane osteotomy runs in an oblique direction from the superior margin of the axial osteotomy to the anterior femoral cortex. After that, the first cut of the anterior plane osteotomy is performed with an oscillating saw from medial to lateral with the saw blade slightly angled towards the floor. Both the medial and contralateral lateral cortex is completely cut through. For derotation osteotomy, the target correction rotation angle calculated in preoperative planning and was marked using two Kirschner wires intra-operatively under the fluoroscopic guidance. The lateral cortex was cut completely and two Steinmann nails were placed proximally and distally to the osteotomy site to allow the distal femur external rotated to the predetermined angle. After that, a medial-based anterior wedge is produced by a second anterior saw cut inferior to the first wedge. The wedge is removed, and the gap created by anterior osteotomy is closed by rotating residual fragments using the two Schanz screws.

**Fig. 1:**
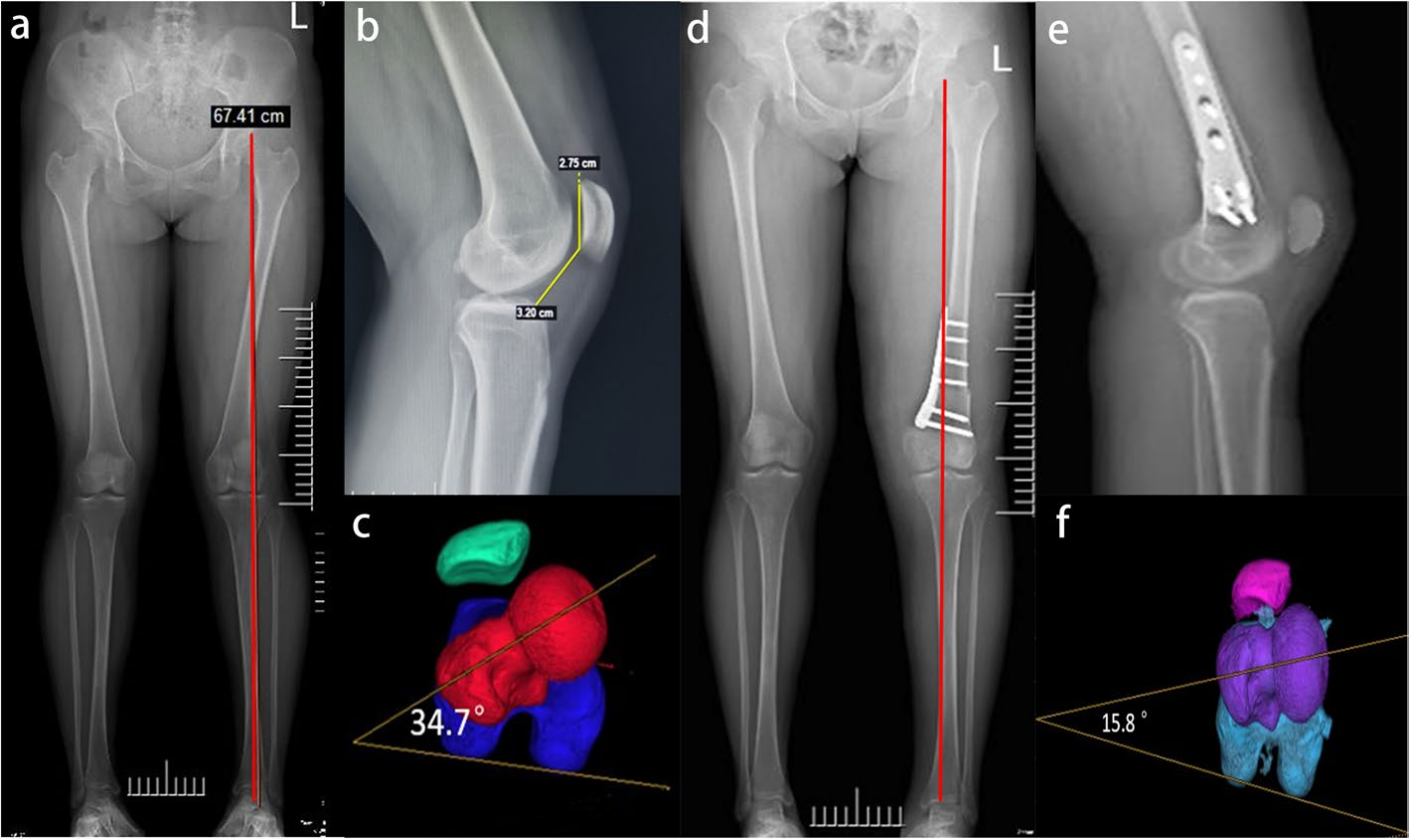
Genu valgum deformity with recurrent patellar dislocation of a 19-year-old female. **a** Preoperative full-leg standing anteroposterior radiographs showed knee valgus deformity. **b.** Preoperative lateral radiograph. **c.** The preoperative femoral anteversion angle was increased to 34.7°. **d**. Postoperative full-leg standing anteroposterior radiographs showed neutral alignment. **e.** Postoperative lateral radiograph showed stable fixation with a Tomofix-locking plate. **f.** CT scans postoperatively showed an improvement in femoral anteversion angle

After that, patellar tracking was re-evaluated under arthroscopic visualization after osteosynthesis with Tomofix-locking plate fixation (Depuy Synthes, Umkirch, Germany). Of note, it is important to place the Tomofix-locking plate fixation in a position where they do not impede the later positioning of the femoral tunnel of MPFL. Typical case was presented in Fig. 1**.**

#### MPFL reconstruction

The MPFL double-bundle anatomical reconstruction using the semitendinosus tendon autograft was performed in all patients and our surgical technique was similar to Schoettle et al. [16]. Two bony grooves were drilled in the patellar medial edge, which were placed into the center and the upper inner corner of the patellar medial edge, respectively. The graft fixed in patella side by two anchors (Andover, MA, USA for Smith &Nephew) inserted into the bony grooves. Subsequently, a femoral tunnel was made at the femoral insertion site of the MPFL, and the graft was fixed by a bioabsorbable interference screw with the knee in 20° to 30° of flexion. Under C-arm guidance, the proper point of the femoral insertion site of the MPFL was identified at the Schoettle point and confirmed using a graft isometric assessment to avoid increased graft tension around 20° of flexion [17, 18]. The mobility of the patella was evaluated to validate that the graft was no tight, and the ROM was examined by cycling the knee from full extension to full flexion.

#### Rehabilitation protocol

All patients started functional exercises within 24 h post-operation, including active circum-movements of ankle and isometric quadriceps muscle training. Patients were instructed to begin partial weight-bearing with crutches for the first 4 weeks, and strengthening exercise of vastus medialis muscle was encouraged. Full-weight bearing without limitation was allowed at 6–8 weeks postoperatively.

#### Functional scores evaluation

Knee function was evaluated using range of motion (ROM) and functional scores, including visual analogue scale (VAS), Kujala score [19], International Knee Documentation Committee (IKDC) score [20], Lysholm score [21], and Tegner activity score [22] were used to evaluate knee function preoperatively and at the last follow-up after surgery.

#### Radiographic assessment

Preoperative radiological analysis prior to surgery, including full-leg standing anteroposterior (AP) radiographs of lower extremity, lateral views, and standardized hip-knee-ankle computed tomography (CT) scans were used to evaluate the mechanical lateral distal femoral angle (mLDFA), anatomical femorotibial angle, mechanical axis, trochlear dysplasia, TT-TG distance, patellar height, and tibial and femoral torsion deformities in the axial plane.

The mLDFA was measured on the full-leg standing AP radiograph and was defines as a lateral angle between a line connecting the center of the femoral head to the center of the knee and a line tangent to distal femoral condylar. Trochlear dysplasia and TT-TG distance were evaluated by axial CT images. The TT-TG distance was measured between the two parallel lines that perpendicular to the posterior condylar tangents on superimposed axial slices: a line passed the most cephalad point of the tibial tubercle and a line passed through the deepest point of the trochlear groove [8]. Caton-Deschamps index (CDI) was measured on lateral radiographs to evaluate the patellar height [23].

**Fig. 2:**
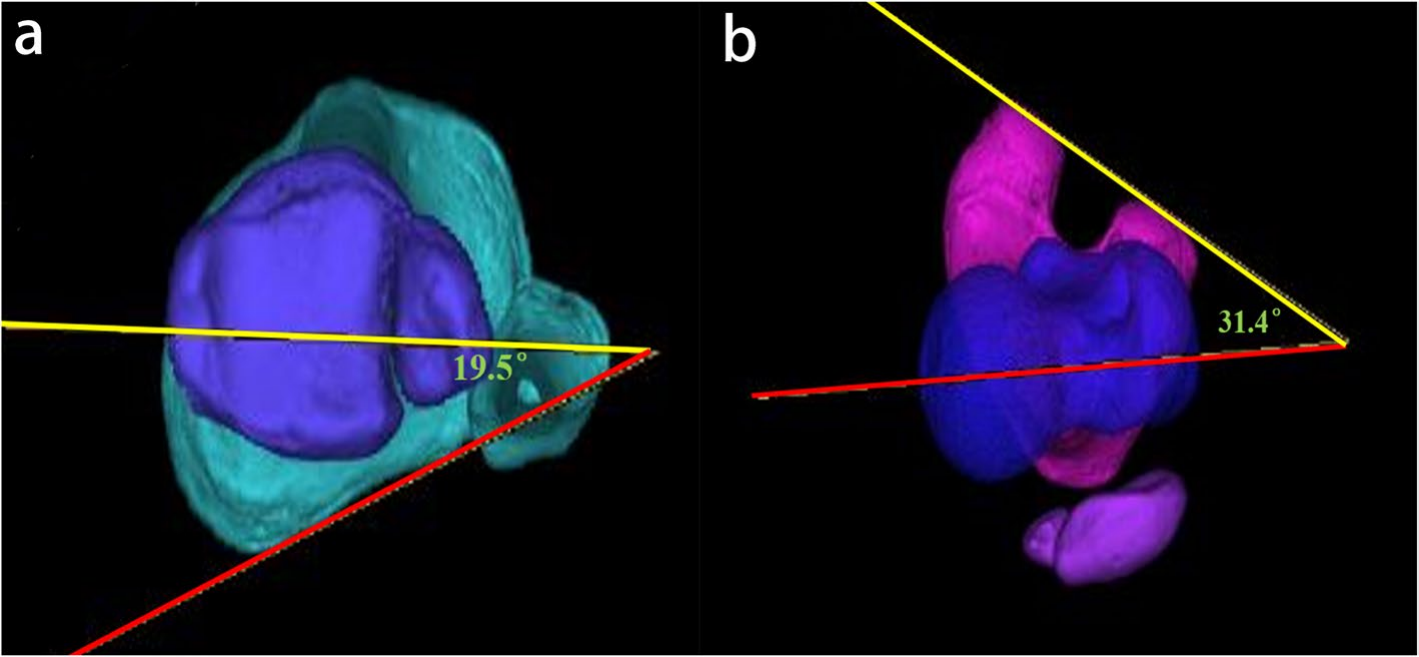
Measurement of torsional parameters of the lower extremity on 3-dimensional computed tomography. **a.** The tibia external rotation was defined as the angle between the line connecting the posterior tibial condyles and the line connecting the midpoints of the medial and lateral malleoli. **b.** The femoral anteversion angle was defined as the angle between femoral neck axis (red line) and posterior condylar of distal femoral axis (yellow line)

Tibial and femoral torsion deformity was measured on reconstructed 3-dimensional axial CT images utilizing the method described by Takagi et al. [24], as shown in Fig. 2. Tibial torsion was defined as the angel between a line connecting the posterior tibial condyles and a line connecting the midpoints of the medial and lateral malleoli. The FAA was defined as the angle between a line connecting the midpoint of the femoral neck and the center of the femoral head and a line connecting the most posterior points of the medial and lateral femoral condyles.

#### Statistical analysis

Statistical analysis was performed using the SPSS software (version 24.0, Armonk, NY, USA for SPSS software package, IBM Corp.). As for continuous variables, the Kolmogorov-Smirnov test was first applied to test normality. Clinical and radiographic results were shown as the mean and standard deviation (SD). Due to the normal data distribution, paired *t* test was used to compare the differences in the pre- and post-operative clinical and radiographic outcome data. For all tests, *p* values < 0.05 were considered statistically significant.

## Results

### Patient demographics

A total of 13 patients (13 knees) who underwent double-bundle anatomical MPFL reconstruction combined with biplanar supracondylar FDO were included in the study. Of the 13 patients, 4 cases were males and 9 cases were females. The average age of the included patients at the time of surgery was 18.7 years (range, 15–29 years). The average body mass index (BMI) was 25.7 ± 3.6 (range, 21.8–31.2). The average follow-up period was 26.7 months (range, 24–33 months) (Table 1**).**

**Table 1:** Patient demographics

Variable	Value
Number of patients	13
Age, years (range)	18.7 (15–29)
Sex, male/female	4/9
Side, left/right	5/8
BMI, kg/m^2^ (range)	25.7 ± 3.6 (21.8–31.2)
Follow-up period, months (range)	26.7 (24–33)

*BMI* body mass index

### Clinical outcome

ROM and functional scores after surgery were shown in Table 2. The average VAS score for pain significantly decreased from 4.81 ± 2.13 preoperatively to 1.83 ± 1.47 at the final follow-up (*p* < 0.001). The average Kujala score improved significantly from 57.48 ± 8.76 preoperatively to 87.43 ± 4.25 at the final follow-up (*p* < 0.001). The average IKDC score improved significantly from 51.42 ± 8.36 preoperatively to 83.59 ± 7.27 at the final follow-up (*p* < 0.001). The average Lysholm score improved significantly from 59.85 ± 9.71 preoperatively to 83.88 ± 6.45 at the final follow-up (*p* < 0.001). The average Tegner activity score improved significantly from 2.2 ± 1.3 preoperatively to 4.5 ± 1.8 at the final follow-up (*p* < 0.05).

**Table 2:** Comparison of pre- and post-operative knee functional scores and pain

Variables	Pre-operative	Post-operative	*p*-value
ROM	128.90 ± 5.60	133.20 ± 7.60	0.114
VAS pain	4.81 ± 2.13	1.83 ± 1.47	< 0.001^*^
Kujala score	57.48 ± 8.76	87.43 ± 4.25	< 0.001^*^
IKDC score	51.42 ± 8.36	83.59 ± 7.27	< 0.001^*^
Lysholm score	59.85 ± 9.71	83.88 ± 6.45	< 0.001^*^
Tegner activity score	2.2 ± 1.3	4.5 ± 1.8	< 0.05^*^

*ROM* range of motion, *VAS* visual analog scale, *IKDC* International Knee Documentation Committee

*Significant difference compared to preoperatively

### Radiographic outcomes

Radiographic outcomes after surgery are shown in Table 3. Compared with pre-operation, the mLDFA, aFTA, and mechanical axis showed statistically significant improvement following the combined surgery (*p* < 0.001), while the CDI did not change significantly after surgery (*p*>0.05). The mean TT-TG distance significantly decreased from 19.63 ± 3.21 mm to 13.29 ± 2.78 mm (*p* < 0.001), The pre-operative FAA was 32.77° ± 3.78°, and it was 19.08° ± 3.14° at the final follow-up (*p* < 0.05).

**Table 3:** Comparison of pre- and post-operative radiological parameters

Variables	Pre-operative	Post-operative	*p*-value
mLDFA, degree	82.72 ± 3.27	88.63 ± 2.35	< 0.001^*^
aFTA, degree	166.32 ± 2.14	175.46 ± 2.39	< 0.001^*^
Mechanical axis, degree	6.6 ± 1.2	0.2 ± 0.7	< 0.001^*^
TT-TG (mm)	19.63 ± 3.21	13.29 ± 2.78	< 0.001^*^
CDI	1.11 ± 0.13	1.15 ± 0.08	0.354
Femoral anteversion, degree	32.77 ± 3.78	19.08 ± 3.14	< 0.001^*^

Values are presented as mean ± standard deviation *n.s* not significant, *CDI* Caton-Deschamps index, *mLDFA* mechanical lateral distal femoral angle, *aFTA* anatomical femorotibial angle,*TT-TG* tibial tubercle to trochlear groove

*Significant difference compared to preoperatively

### Complications

No cases developed wound infection, soft tissue irritation, and recurrence of patellar subluxation or dislocation during the follow-up period after surgery. Bone healing at the osteotomy site was achieved in all cases, and all patients regained full extension and flexion and no limited range of motion was observed.

## Discussion

In this retrospective pilot study, the most important finding in the present study is that the treatment of RPD with an increased FAA (> 25°) and genu valgum using MPFL reconstruction combined with supracondylar biplanar FDO is effective, with no reported re-dislocation of the patella. Through the abovementioned combined surgery, radiological correction of the patellofemoral instability, excessive femoral anteversion and genu valgum could be achieved, and significant improvements of clinical outcomes could be obtained. Generally, the presence excessive femoral anteversion and genu valgum are known risk factors for patellar dislocation [8, 9], and cause many clinical manifestations, including anterior knee pain, patellofemoral instability, and gait disturbance [25]. Therefore, surgery treatment of RPD aims to correct maltracking of the knee extensor mechanism, which is benefit for restoring a normal mechanical environment of the patellofemoral joint in this population.

Patellar instability associated with genu valgum treated by supracondylar distal femoral osteotomy have been reported in several studies [26–28]. Nha et al. [10] demonstrated the satisfactory improvement of knee function of 14 patients (23 knees) who underwent closing-wedge distal femoral osteotomy without MPFL reconstruction. Similarly, Swarup et al. [11] demonstrated that lateral opening wedge distal femoral osteotomy combined with lateral retinacular release yield satisfactory clinical results in this population. However, the clinical significance and potential advantages of MPFL reconstruction combined with biplanar supracondylar FDO procedure in RPD with increased FAA and genu valgum have not yet been identified. In this retrospective study, MPFL reconstruction combined with biplanar supracondylar FDO procedure achieved significant functional improvement after surgery in knee function scores (VAS, Kujala score, IKDC score, Lysholm score, and Tegner scores) and satisfactory radiographic outcomes (FAA, TT-TG, mLDFA, aFTA, and mechanical axis) in patellar instability with increased FAA and genu valgum, and no recurrence of dislocation cases had been found within the follow-up period.

Several orthopaedic surgeons have emphasized the role that osseous deformities of the axial and coronal plane acts as a significantly higher risk factor for patellofemoral maltracking. Dejour et al. [14] identified that patients with patellofemoral instability had a higher value of FAA comparted to healthy controls (15.6 vs. 10.8) following CT evaluation. Similarly, Zhang et al. [29] have reported that the adverse effects of increased femoral internal torsion on reconstructed MPFL, especially in patients when the FAA greater than 30°, which could be partially explained by the fact that the excessive lateralizing force vector acting on the patella due to the increased Q angle [25]. Recently, biomechanical studies further demonstrated that the adverse effect of isolated MPFL reconstruction for patellar instability associated with increased FAA. Kaiser et al. [30] revealed that isolated MPFL reconstruction for patellar instability is insufficient for higher degrees of FAA, which indicated that increased FAA may result in a persistent lateral force vector on the patella.

Due to these abnormal biomechanics of osseous deformity, it is vital to identify these underlying predisposing risk factors and to early make intervention for RPD. Despite isolated anatomical MPFL reconstruction is considered to be a standard treatment for patellofemoral instability with satisfactory results [31, 32], subsequent studies have demonstrated that a high rate of subjective dissatisfaction in patients with increased femoral internal torsion [33]. Supracondylar FDO as an isolated procedure has been shown good clinical outcomes for RPD with increased FAA [34, 35]. However, isolated FDO procedure at the distal femur may increase the risk of graft failure when ignoring the correction of the knee valgus deformity, because the laterally-oriented vector forces applied to patellofemoral joint which can result in excessive tension into the reconstructed MPFL graft [5, 9].

Recently, there has been a great focus of investigating the effect of derotation femoral osteotomy on the changes of coronal alignment. Nelitz et al. [36] reported that FDO procedure tend to result in an increased valgus angulation in the frontal plane due to a decreased mLDFA. Similarly, Konrads et al. [37] also identified that supracondylar femoral external osteotomy would lead to valgus deformity of the coronal limb alignment, which may be attributed to the reorientation of the femoral antecurvature and the femoral neck. Despite a biplanar supracondylar DFO has been performed in the correction of valgus deformity and excessive femoral internal torsion simultaneously, none of this group of patients showed signs for delayed union or non-union of the osteotomy, which was comparable to Imhoff et al. [34] on a combined varus and external rotation producing distal femoral osteotomy.

There are several limitations in this study. The main limitation of this study is the small sample. It has to be highlighted that patients underwent this combined surgery have to be selected depending on rigorous inclusion criterions. In addition, the retrospective study design of the present study and the small number of patients included should be considered when interpreting our results. Future studies with larger patient cohorts are needed to further confirm the clinical outcome of this combined surgery. Therefore, as this was a pilot study and only a limited number of patients were available, no power analysis was performed. Furthermore, a missing comparative group of patients who treated by other surgical techniques. However, considering that the significant functional improvement and absence of re-dislocation, FDO combined with MPFL reconstruction may be a treatment option for RPD with increased FAA (FAA > 25°) and genu valgum.

Finally, second-look arthroscopic evaluation was not performed to evaluate the changes of trochlear and retropatellar cartilage.

## Conclusion

MPFL reconstruction combined with biplanar supracondylar FDO is a safe and reliable treatment for RPD with increased FAA and genu valgum, which demonstrated a satisfactory clinical and radiographic results in the short-term follow-up.

## Data Availability

All the data and material involving this article will be available upon request by send an e-mail to the first author.

## Abbreviations

MPFL: Medial patellofemoral ligament
FDO: Femoral derotation osteotomy
RPD: Recurrent patellar dislocation
FAA: Femoral anteversion angle
CT: Computed tomography
mLDFA: Mechanical lateral distal femoral angle
TT-TG: Tibial tubercle-trochlear groove
IKDC: International knee documentation committee
VAS: Visual analog scale
AP: Anteroposterior
CDI: Caton-deschamps index
SD: Standard deviation
BMI: Body mass index
